# A proposed metric for assessing the measurement quality of individual microarrays

**DOI:** 10.1186/1471-2105-7-35

**Published:** 2006-01-23

**Authors:** Kyoungmi Kim, Grier P Page, T Mark Beasley, Stephen Barnes, Katherine E Scheirer, David B Allison

**Affiliations:** 1 Department of Biostatistics, Section on Statistical Genetics, University of Alabama at Birmingham, Birmingham, Alabama, USA; 2 Department of Pharmacology and Toxicology, University of Alabama at Birmingham, Birmingham, Alabama, USA; 3 Heflin Center for Human Genetics, University of Alabama at Birmingham, Birmingham, Alabama, USA

## Abstract

**Background:**

High-density microarray technology is increasingly applied to study gene expression levels on a large scale. Microarray experiments rely on several critical steps that may introduce error and uncertainty in analyses. These steps include mRNA sample extraction, amplification and labeling, hybridization, and scanning. In some cases this may be manifested as systematic spatial variation on the surface of microarray in which expression measurements within an individual array may vary as a function of geographic position on the array surface.

**Results:**

We hypothesized that an index of the degree of spatiality of gene expression measurements associated with their physical geographic locations on an array could indicate the summary of the physical reliability of the microarray. We introduced a novel way to formulate this index using a statistical analysis tool. Our approach regressed gene expression intensity measurements on a polynomial response surface of the microarray's Cartesian coordinates. We demonstrated this method using a fixed model and presented results from real and simulated datasets.

**Conclusion:**

We demonstrated the potential of such a quantitative metric for assessing the reliability of individual arrays. Moreover, we showed that this procedure can be incorporated into laboratory practice as a means to set quality control specifications and as a tool to determine whether an array has sufficient quality to be retained in terms of spatial correlation of gene expression measurements.

## Background

Gene expression microarrays are a powerful tool used in molecular biology and genetics for understanding gene expression change in biological processes under normal and pathological conditions [1]. Intensity measurements of gene expression are associated with significant variations as a result of the complex and multi-stage processing involved in microarray experiments. Beyond the variability that may be introduced during the fabrication of arrays as a result of print substrate quality and printing pin anomalies, several processing steps – mRNA sample extraction, amplification and labeling, hybridization, and scanning – may introduce substantial variation in measurements [2]. Although several studies have characterized the potential impact of these latter sources of variation on measurements of gene expression [2-4], methods for assessing the physical measurement quality of individual microarrays are not widely available. If technical replicates for a biological case are available, the degree of concordance between technical replicates can be used as an index of reliability [5]. However, when only one technical replicate is available per biological case, this information cannot be generated. Even in situations where two technical replicates are available, at least one of the replicates must have adequate quality, preferably based on objective rather than subjective evidence, to be used to determine the degree of concordance between the two technical replicates. Therefore, it is important to seek an alternative objective metric by which we can judge the quality of individual microarrays.

Ideally, all intensity measurements should be randomly expressed without any systematic or spatial patterns across an array. In other words, the geographic location of each gene expression measurement on an array should not be related to its intensity level. The intensity level of the same gene should be constant no matter where the gene locates on the array, meaning that geographic locations do not determine intensity levels. However, this assumption is not always true. For example, we assume that all arrays are produced through routine microarray procedures according to the manufacturing protocols or widely accepted academic protocols. Some measurement artifacts may create patterns of hybridization that depend on the geographic positions of these artifacts on arrays. An example would be the case of an imbalanced (uneven) hybridization across an array. An array may have a smudgy high background signal or "strain" on certain geographic regions, creating a pattern of hybridization dependent on geographic position. Another extreme example would be the case of physical scratches caused by coverslip removal or finger imprints on an array. In the absence of such artifacts, gene expression levels should show no unusual spatial pattern or bias across the microarray's surface other than systematic patterns due to the array design in the manufacturing of chip. Thus, if we compute an index for an array by which a geographic position on the specific microarray's surface predicts its intensity level of gene expression, then this index may provide information about the summary of physical reliability of an individual array based on spatial correlation.

To create such an index, it is necessary to clarify the meaning of independence between two variables. Herein, we define that two variables are *geographically independent *if knowing the value of one variable provides no information about the location of the other variable, and vice versa. We define the geography index as a quantitative measure of the degree of geographical independence between intensity measurements and their geographic locations on an array. Presumably, higher geography index values indicate no significant spatial correlation between intensity measurements and their physical locations on an array. Arrays with higher geography index values are considered to be more reliable. Moreover, if two replicate arrays have high, constant geography index values, then the two arrays are considered physically reproducible in terms of spatial correlation. Therefore, in this study we examine the spatial correlation between gene expression levels and their geographic locations on an array. We propose a statistical approach by regressing measurements on a third degree of the polynomial response surface model of the microarray's Cartesian coordinates to calculate a geography index. To illustrate the utility of geography index and evaluate its validity, we first conducted tests with a series of real arrays from several microarray experiments and with a set of simulated arrays. Second, we compared two different microarray platforms – spotted cDNA arrays and Affymetrix chips – to evaluate whether this method could be applied generally for microarray technology. From a practical point of view, this metric allows investigators to determine the quality of individual arrays. Further studies will be needed with larger sets of microarray data to develop benchmark quality values for this geography index so that threshold values can be set to determine whether an array has sufficient quality to be retained for further downstream analysis such as identification of differentially expressed genes.

## Results

### Assessing the reliability of measurements using the geography index

**Figure 1:**
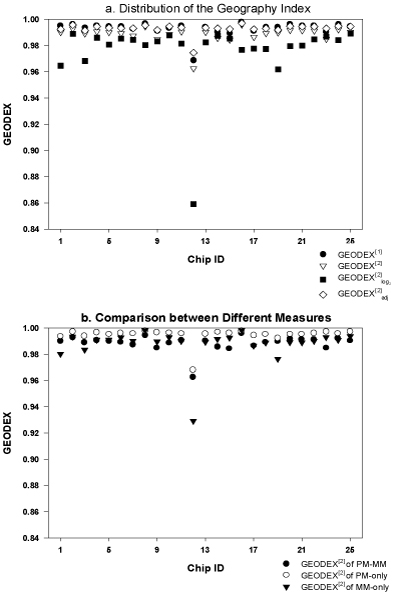
**Distributions of geography index (GEODEX) values of individual chips of experiment A**. a. Black circles and white upper triangles represent GEODEX computed by model (1) and model (2), respectively. Black rectangles and white diamonds represent GEODEX computed by model (2), using measurements in the log scale on base 2 and trimmed measurements after removing four blocks on the corners, respectively. b. For comparisons between different measures of gene expression, black circles, white circles, and black upper triangles represent GEODEX of difference (PM-MM) measurements of pairs of the perfect match (PM) and mismatch (MM) probes, of PM-only measurements, and of MM-only measurements, respectively. GEODEX were computed by model (2) (N_B _= 36). X-axis indicates a chip identification number and Y-axis indicates the degree of GEODEX.

**Figure 2:**
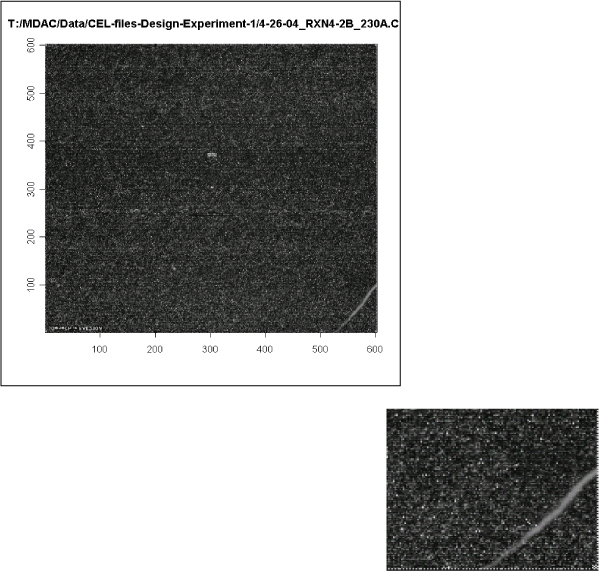
**Image of Affymetrix chip 12 of experiment A**. The bottom area that was damaged by technical mistakes during scanning on the right corner of chip 12 is shown enlarged.

Twenty five Affymetrix™ arrays of experiment A were used to evaluate the utility of geography index, which served as a predictor of the physical quality of arrays. For the assessment of reliability of arrays, the global geography index (GEODEX) for individual chips was calculated by fitting all intensity measurements (perfect match [PM], mismatch [MM], and PM-MM difference intensities) to model (1). Results are summarized in Table 1. The distribution of GEODEX (black circles) of all chips is depicted in Figure 1(a). The degrees of GEODEX^[1] ^were almost constant, except for Chip 12, which appeared to have more spatial correlation than others. It was evident that Chip 12 possessed a systematic pattern or irregular behavior dependent on geographic locations across the array. For this reason, we visually inspected each individual chip and found that Chip 12 had been damaged by careless handing during the scanning procedure and contained a small artifact across the array (see Figure 2). This result demonstrated that the proposed metric was capable of pinpointing chips that contained artifacts on arrays. However, it was possible that the bias of the small artifact might not influence the overall spatial variation. As a result, the relative amount of variance due to the artifact was underweighed. Hence, we studied the spatial variability of arrays while taking blocks into account as a regressor of block-dependent variance. We calculated robust GEODEX of individual chips using model (2). The results showed that the regressor (block) increased the overall spatial correlation and reduced the degree of GEODEX^[2] ^(see Figure 1(a)). Chip 12 still displayed the lowest geography index.

For the sensitivity of block size on results of model (2), we compared results from three different block sizes: 1204 × 1204 features per block (N_B _= 9), 602 × 602 features per block (N_B _= 36), and 301 × 301 features per block (N_B _= 144), where N_B _is the total number of blocks per chip. As shown in Table 1, GEODEX for all the three different sizes appeared to have the same trend, and GEODEX became smaller as the number of defining blocks per chip increased. Hence it is possible to make fine distinctions between more reliable chips and less reliable chips when a chip is defined with a larger number of blocks. However, more work is needed to define appropriate block sizes.

**Figure 3:**
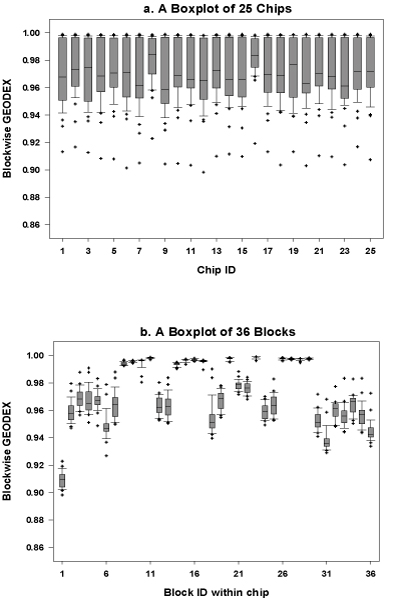
**Distributions of blockwise GEODEX**. a. A boxplot of 25 chips: Each box shows the distribution of blockwise GEODEX for 36 blocks per chip (i.e., N_B _= 36). The X-axis indicates a chip identification number. b. A boxplot of 36 blocks: Each box shows the distribution of blockwise GEODEX for 25 chips per block. The X-axis indicates a block identification number within the chip. Crosses represent outliers.

For most measurements, two technical measurement replicates per biological case should be highly correlated. On the other hand, outliers or problematic blocks distort variances. Most outliers are likely to be due to measurement errors and, therefore, tend to cause not only low reliability but also poor quality of arrays. Those outliers can be detected by noting whether the observed intensity value is within the possible range of values. Relative to this point, we studied the distribution of blockwise GEODEX to identify blocks that displayed significant deviation relative to other blocks. Those highly deviant blocks could be outliers and strongly influence the spatial correlation. Blockwise GEODEX_*Bij *_for each block *B*_*ij *_(*j *= 1, 2, ..., 36) on each slide *i *was calculated using model (3). Block effects varied from chip to chip, and each chip had outliers (see Figure 3(a)). Blocks 1, 6, 31, and 36 on the four corners of the arrays tended to have the lowest GEODEX_*Bi *_values, indicating that these blocks were more likely outliers and caused the edge effects (see Figure 3(b)). This may not be unusual in microarray experiments because of unstable conditions of hybridization around the boundaries of slides. Moreover, there was a strong trend that the non-edge blocks (i.e., blocks 8–11, 14–17, 20–23, and 26–29) had higher blockwise GEODEX values and smaller blockwise GEODEX variance for all the arrays. This finding suggested that the measurements in those non-edge blocks had less spatial correlation between the intensity levels and their locations on the arrays and were less affected by the spatial effects. This implied that a certain amount of edge effects would be an impediment to assessing the quality of measurements. In this case, local normalization of measurements for spatial effects, such as local normalization based on loess nonparametric regression, may be helpful to relieve issues related to edge effects [12,13].

To reduce the effects of outliers on overall spatial variance, we trimmed the distribution of measurements by removing a small number of blocks that had high spatial correlation. We used the trimmed measurements to recalculate a global geography index, GEODEX^[2]^_adj_, using model (2). We compared GEODEX^[2] ^obtained using the entire array including all the measurements with GEODEX^[2]^_adj _obtained using the trimmed array that discarded blocks having lower blockwise GEODEX (see Figure 1(a)). When we discarded edge blocks 1, 6, 31, and 36 (i.e., those that had high spatial correlations), the magnitude of GEODEX^[2]^_adj _improved. This result led to two findings: 1) the measurements around the edges exaggerated the overall spatial correlation and 2) the outliers deflated the magnitude of GEODEX. Therefore, investigators interested in unraveling the spatial effects of outliers are well advised to perform an additional analysis to evaluate whether results change when highly deviant blocks or outliers are excluded from the study. Note, however, that it is unwise to exclude such outliers when the interest is in cross comparisons between arrays for the purpose of a chip's quality check.

### Assessing the validity of the geography index as a predictor of reproducibility

**Figure 4:**
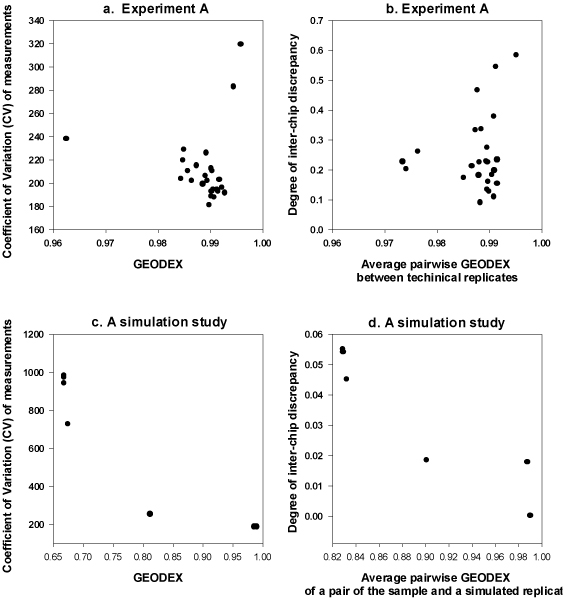
**Assessing the validity of geography index**. Scatter plot a (c) shows the relationship between GEODEX and the coefficient of variation (CV) of measurements for experiment A (a simulation study). Scatter plot b (d) presents the relationship between pairwise GEODEX computed by equation (5) and the degree of inter-chip discrepancy computed by equation (4) for every pair of technical replicates for biological samples in experiment A (for every pair of the sample array and simulated replicates for a simulation study). GEODEX was computed by model (2).

Figure 4 depicts scatter plots of the coefficient of variation (CV) and GEODEX and scatter plots of the degree of inter-chip discrepancy of all pairs containing technical replicates against pairwise GEODEX calculated by equations (4) and (5), respectively. Dataset A showed that most chips formed a cluster of low CV and high GEODEX (Figure 4(a)) and a cluster of low inter-chip discrepancy and high pairwise GEODEX (see Figure 4(b)); both clusters entailed high correlation between the technical replicates and implied high reproducibility of the arrays in a broad sense. However, the magnitude of inter-chip discrepancy was not sufficient to render the degree of spatial concordance and/or discordance between two technical replicates. Hence, it was hard to provide convincing evidence that GEODEX could predict the spatial concordance between two technical replicates to assess reproducibility with respect to the spatiality feature of measurements on arrays. There may be a feasible explanation for this finding. If the spatial effects between two technical replicates were sufficiently different to discriminate the two arrays, GEODEX would predict reproducibility between the two technical replicates. In contrast, if there was no marginal variation of spatial effects between the technical replicates (i.e., no significant inter-chip discrepancy), it would be difficult for GEODEX to predict the reproducibility between the two replicates. Thus this approach may not be capable of assessing the reproducibility between technical replicates for the same biological sample in the absence of substantial spatial effects on the arrays.

Alternatively, we conducted a simulation study to explore the validity of GEODEX as a predictor of reproducibility of arrays based on spatial concordance. We randomly took a chip from experiment A as a "sample" array and added systematic noise only to block 15 located in the middle of array. Systematic bias was simulated under eight different scenarios: 1) added systematic bias from a standard normal distribution (e.g., *k *- N(0, 1)), in which the randomness across the array was not affected by artificial bias; 2) decreasing the intensity values by subtracting a constant (e.g., *k *- 100); 3) increasing the intensity values by multiplying by 2 (e.g., k *2); 4) multiplying by 10 (e.g., k *10); 5) multiplying by 100 (e.g., k *100); 6) multiplying by the standard deviation (SD) of the measurements on the sample chip (e.g., k *SD); 7) multiplying by two SDs (e.g., k * 2 SD); and 8) multiplying by three SDs (e.g., k * 3 SD). We hypothesized situations in which the background hybridization signal should be uniform across the array; however, small artifacts, such as smudges or scratches or imbalanced hybridization, changed the gene expression levels on block 15. We created eight simulated technical replicates plus the sample array with identical measurements except for those on block 15, maintaining the randomness property of the arrays. Introducing bias on block 15 inflated the overall variances of the individual chips even though the first two cases had very little influence on the overall variances and were less informative than the other cases. However, block 15 was the only source of between-chip variance and leveled the degree of inter-chip discrepancy between the sample array and other simulated arrays. We were interested in examining what would happen to the relationship between the inter-chip discrepancy and pairwise GEODEX of the eight pairs of the two replicates. We calculated GEODEX^[1] ^and GEODEX^[2] ^for the simulated chips using models (1) and (2), respectively. Table 2 summarizes the results. The relationship between GEODEX^[2] ^and the CV of the intensity measurements is depicted in Figure 4(c). In the presence of spatial effects by block 15, GEODEX and CV had a negative relationship, indicating that the degree of geographic independence increased as CV decreased. Note that the spatial variation dependent on the intensity measurements of the geographic locations was proportional to the overall variance of the measurements on the arrays. The excessive spatial correlation reflected more measurement error of the measurements on the arrays and thus induced less reliability of the arrays. A scatter plot of the degree of inter-chip discrepancy of the eight pairs of the sample chip and every simulated replicate against pairwise GEODEX is depicted in Figure 4(d). As would be expected, the increase in pairwise GEODEX was opposite to the decrease in the inter-chip discrepancy. This simulation study affirmed the validity of the hypothesis that GEODEX is capable of predicting the reproducibility of measurements when there is enough inter-chip spatial variance between two replicates. From comparisons of GEODEX^[1] ^with GEODEX^[2]^, we found that GEODEX^[2] ^was affected by variation from a small cluster of extremely expressed genes or a small artifact, and the bias of the small artifact on block 15 exaggerated the overall spatial correlation. Therefore, model (2) is more effective for estimating spatial correlation than model (1) in situations in which block-to-block differences across an array are significant.

**Figure 5:**
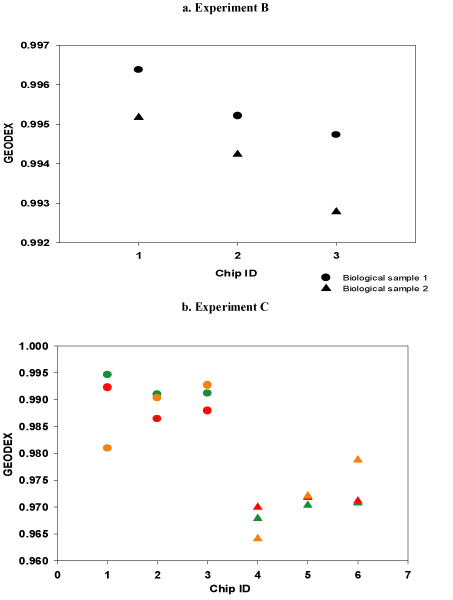
**Distributions of GEODEX of individual chips of experiments B and C**. a. Each shape stands for each biological sample: circles for biological sample 1 and triangles for biological sample 2. b. For a comparison between different microarray platforms, circles and triangles represent Affymetrix oligonucleotide arrays (chips 1–3) and cDNA arrays (chips 4–6), respectively, and orange, green, and red represent Cy5/Cy3, Cy3-only, and Cy5-only measures, respectively. GEODEX was computed by model (2).

The findings from experiment A were commonly observed when the total six chips (two biological replicates by three technical replicates) of experiment B were analyzed using model (2): chip 3 of biological sample 2 had the lowest GEODEX, suggesting that this chip had a higher spatial correlation between the intensity levels and their locations on the array than others (Figure 5(a)); and the spatial concordance between technical replicates within a biological case was higher than that between technical replicates across biological cases (data not shown).

Despite the limitations of GEODEX as a predictor of reproducibility of technical replicates in general, GEODEX could be used to assess the reproducibility depending on spatial patterns. We supposed the two most interesting cases: 1) one technical replicate (hereafter referred to as shifted) had all its expression measurements shifted by 10 x-units relative to the other technical replicate (referred to as non-shifted); the x-axis coordinates for the shifted were changed by 10 units and the y-axis coordinates were unchanged and 2) one technical replicate had all its expression measurements shifted by both 10 x-units and 10 y-units relative to the other technical replicate; both the x-axis and y-axis coordinates for the shifted were changed by 10 units. In these cases, the Pearson correlations between the two replicates (non-shifted and shifted) were less than .33 (data not shown), suggesting that the replicates had a low correlation due to inconsistency between corresponding measurements. However, the two replicates had the exactly same GEODEX value, indicating that the two replicates were reproducible in terms of their spatial correlations. Hence GEODEX can be used to assess reproducibility of technical replicates for such particular cases in which one replicate is shifted vertically or horizontally systematically during printing or scanning as illustrated above.

### Effects on results of the violation of assumptions

We examined whether the violation of assumptions within the models influenced the analyses. Because the standard deviation (SD) of measurements of gene expression was proportional to the mean value (see Table 1), dataset A was log-transformed to normalize the distribution with residual errors that had the same variance for all measurements. Then we recalculated GEODEX using model (2) and compared GEODEX^[2] ^of the untreated data with GEODEX^[2]^_log2 _of the log-transformed data (see Figure 1(a)). GEODEX^[2]^_log2 _was smaller than GEODEX^[2]^, suggesting that the log-transformation provided a better fitting polynomial model with a larger R^2 ^that implied small error or variability in the prediction of spatial correlation. In contrast, the untreated data had a poor fitting polynomial model in which R^2 ^was smaller than that of the log-transformed data. Thus, this provided a less accurate estimation of spatial variation of measurements associated with their geographic locations on an array.

The use of appropriate transformations may make the proposed method more plausible [14]. However, the two distributions of GEODEX of the two data treatments had similar patterns in general. After log-transformation the only significant difference was that a range of GEODEX values varied and discriminated from each other more remotely. Therefore, without a loss of significance, we chose to retain data in the original scale for the sake of simplicity. Nevertheless, it is noteworthy that the proposed metric for assessing the geographic independence of measurements on geographic locations was not affected exclusively by the distributions of measurements. Therefore, the proposed approach perhaps can relax distributional assumptions.

### Comparison between different measures of gene expression

For Affymetrix chips we calculated GEODEX with PM-only and MM-only intensities using model (2). Scatter plots showing the distributions of GEODEX for PM-only and MM-only intensity measures are depicted in Figure 1(b). The results of these analyses confirmed that the intensity levels of chip 12 showed less geographic independence from the geographic locations on the chip. However, the geographic independence appeared to differ depending on which gene expression measure was used. GEODEX^[2] ^of PM-MM and PM-only measurements were close to being parallel. In contrast, GEODEX^[2] ^of MM-only measurements differed from the others. Overall, some interesting findings were observed: 1) It was obvious that the MM-only measurements depended more heavily on their geographic positions on the arrays than did the other measurements. This finding was not surprising because of the additive signals on every 25th MM probe that appeared regularly across array-enhanced systematic patterns on the array surface. 2) For most chips, GEODEX^[2] ^for the three measures were parallel and very close to each other, suggesting that the MM-only measurements detected not only a non-specific binding signal but also a transcript signal (at least partially), as did the PM-only measurements. Also noted was that the PM-MM difference measurements included noise. 3) As one would expect, the PM-only measurements, which did not account for non-specific binding, appeared to have less spatial correlation, suggesting that PM-only is perhaps a more adequate expression measure especially when MM-only has significant spatial effects [15].

### Comparison between different microarray platforms

Experiment C was originally conducted to perform cross comparisons of two different platforms, cDNA spotted arrays and Affymetrix oligonucleotide arrays. In this experiment, two samples of mRNA were labeled with two different fluorophores (Cy5 and Cy3) and co-hybridized onto a glass slide on which PCR products generated from 15,000 thousand clones were immobilized at array positions. Ratios (Cy5/Cy3) of two dye intensities were then calculated. Cy5-only, Cy3-only, and Cy5/Cy3 measures were compared to assess the measurement reliability of arrays. As shown in Figure 5(b), there was a difference between the two platforms: GEODEX of the cDNA arrays was smaller than that of the Affymetrix oligonucleotide arrays, indicating that the Affymetrix oligonucleotide arrays used in this study had less spatial correlation or bias. This seems reasonable because Affymetrix oligonucleotide arrays have random manufacturing systems of ordering features across an array comparing to cDNA spotted arrays placing spots functionally across an array with 32 different printing pins. In addition, it is important to consider different measures of gene expression level. We found that there was no significant difference in dye-dependent spatial bias between two dyes (Figure 5(b)). However, the results showed that taking the ratio of two dye intensities could increase or reduce spatial bias even though the ratio measurements reduced variation in measurements within an array and variance between technical replicates. Therefore, when the ratios of two dye intensities are considered for gene expression analysis, a normalization procedure taking into account spatial variance of expression measurements would be an alternative strategy for removing systematic variations in microarray experiments [12,13].

## Discussion

We have developed a metric for evaluating the physical reliability of individual arrays in terms of a degree of spatial correlation among expression measurements. The proposed GEODEX metric was designed to estimate a degree of the geographical independence between gene expression measurements and their geographic locations on an array. This provided an equivalent measure of spatiality-based physical reliability of an array. Through the analyses of multiple real datasets, we found that GEODEX was useful and valid for assessing the quality of individual chips in terms of spatial correlation. Specifically, 1) GEODEX could be used to check the quality of an individual chip in which there are multiple biological replicates available but no technical replicates per biological case. In particular, it could identify seriously damaged chips or chips with small artifacts leading to poor array quality. Because the array detected in experiment A was so obviously at fault, one could argue that inspecting the arrays visually was at least as effective as using GEODEX. Hence, we will investigate how sensitive the method is to small artifacts that are not visually identified but still lead to poor chip quality in future studies. 2) In the presence of sufficient spatial effects on arrays, GEODEX predicted spatial concordance between technical replicates that could be used as a predictor of the reproducibility of technical replicates. 3) If global GEODEX indicated that some kind of spatial artifacts were on arrays, blockwise GEODEX could lead to suspect locations. Also, edge effects and other systematic biases were detected by conducting analysis of blockwise GEODEX. 4) This method was easy to implement in software with a standard statistical analysis tool. Hence, it is readily accessible to laboratory scientists. Therefore, GEODEX offering a good prediction of physical array reliability in terms of spatial correlation can be installed in laboratories as a quality control monitor to allow investigators to determine whether arrays have adequate quality to be retained as part of a larger data set.

Regarding issues of model choice in analysis, we chose an intermediate cubic polynomial response surface model as a compromise between oversimplifying and overspecifying variables (e.g., to avoid under-parameterizing or over-parameterizing the models) to introduce a novel idea for how to proceed with this proposed approach. However, our approach is not restricted to a particular model and is flexible enough to incorporate into models any necessary data-specific regressors that would provide a best approximation of GEODEX. One can also develop other functional models that establish a relationship between measurements and their geographic locations on an array. To enhance the practical utility of this method, development of more extensive prescription-type procedures searching for best models will be undertaken in future studies.

To calculate GEODEX, we have focused on a number of robust models between local approaches (e.g., blockwise GEODEX) and global approaches (e.g., global GEODEX). These ranged from highly local approaches that regressed measurements to neighboring models to completely global approaches that regressed them to across-all-over models. It appeared that a regressor-dependent model, such as model (2), reasonably predicted a chip's quality. On the other hand, local approaches tended to be more effective when spatiality varied across blocks. For example, a block-by-block analysis could not only detect outliers but could also explore the nature of irregular patterns in the neighborhood of outliers, such as edge effects. In contrast, when local GEODEX across blocks did not change dramatically, global approaches were sensitive enough to assess a summary of the physical reliability of arrays.

Developing benchmark GEODEX threshold values for discriminating between good- and poor-quality arrays for different platforms and manufacturers will require application of the metric against much larger and more diverse data sets than those used in this study. The narrow range of GEODEX across all arrays and chips used in this feasibility study suggests that in general all of these arrays were of good quality. A larger, more complex and diverse quality set of array data would provide a test for such benchmarks. However, in practice, a threshold that determines whether a chip should be retained or discarded very likely varies from laboratory to laboratory because of many factors such as different batches, technicians, chip types, production methods, and experiments. Hence it is difficult to judge whether a specific benchmark for one experiment can be transferred to another experiment. The proposed metric would be interpreted relative to other chips obtained from a single experiment in a given laboratory. However, there are some uses of the GEODEX that are not related to using a threshold for discarding chips. For example, one may wish to assess trends in the quality of chips being produced in a facility. In such cases, the judgment of chip quality is based on the empirical distribution of all GEODEX values for chips from a single experiment and/or across multiple experiments. Alternatively one might want to use the index to assess the level of training and development of a student or technician. The GEODEX metric does not appear to be able to distinguish between variability due to positional effects created during fabrication of chip or array versus those effects that arise during labeling and hybridization and, therefore, the validity of the global claims of superiority of one platform or another in cross-platform comparison.

Normalization is the process of removing biases due to technical variation. However, some results show that biases still remain in the data after normalization even normalization adjustments account for local spatial variation [7,12,13]. Smyth and Speed [16] indeed noted that poor spot qualities could contribute to biases. Thus they suggested that a regression-based normalization method might be improved by incorporating quality weights for individual spots. GEODEX can be used as a quality assessment procedure for spots and chips before normalization to identify the spatial position of less reliable spots on the slide so that a quality weight is assigned according to a degree of spot quality. Gene expression data adjusted by a combination of GEODEX and normalization can be used for downstream analysis such as differential gene expression, and their resulting estimates are expected to be more precise because of a reduction of biases in data.

## Conclusion

Assessment of the physical quality of individual chips is important for gene expression studies to obtain reliable analysis results of differential expression. We discussed the potential of a quantitative metric that could summarize the physical reliability of individual arrays based on spatial correlations among gene expression measurements. We also illustrated the possibility of incorporating this procedure into laboratory practices as a means to set quality control specifications and as a tool to determine whether an array has sufficient quality to be retained.

## Methods

### Measurement experiments

The animal experiments were used in protocols approved by the University of Alabama at Birmingham Institutional Animal Care and Use Committee and the Yale University Institutional Animal Care and Use Committee.

#### Experiment A

##### CNGI experiment

CNGI stands for the Center for Nutrient-Gene Interaction in cancer prevention funded by National Cancer Institute as part of a research program organized by Nutritional Science Group of the Division of Cancer Prevention. The central aim of this research is to test whether components in one's diet establish risk for breast, and possibly prostate, cancer later in adult life. Experiment A included samples taken from eight 21-day-old *Sprague Dawley CD *female rats (Harlan, Indianapolis, IN) exposed to genistein (a soy isoflavone) via their mother's milk. The mothers were fed AIN-76A diet (Harlan-Teklad, Indianapolis, IN) containing 200 mg genistein/kg diet. Young rats were sacrificed at day 21 and the 4th mammary glands were extracted and flash-frozen in liquid nitrogen within 3 min of ex-sanguination. Samples were frozen at -70°C for approximately 90 days. The extraneous fat was removed by dissection, samples were processed in Trizol RNA extraction buffer (Invitrogen Co., Carlsbad, CA), and total RNA was generated using Affymetrix RNA extraction and labeling kits (Affymetix Inc., Santa Clara, CA). The RNA samples were run on Affymetrix GeneChip arrays. The RNA samples were split in half. The first half was labeled and run on a RAE 230A chip and the other half was labeled, split, and run across two RAE 230A chips. Later in the analysis we treated all three subsamples as technical replicates even though they were labeled in two separate reactions. Affymetrix arrays were run in the genomics core facility of the Heflin Center for Human Genetics at the University of Alabama at Birmingham. Images were scanned (2500 scanner; Hewlett-Packard Development Co., Houston, TX) and *.Cel files were generated by MAS 5.0 [6] and used for analyses. During the scanning process, one of the technical replicates (chip 12) for biological sample 4 was found upon visual inspection of the scanned image to have a hair in the path of the scanner. The hair was removed and the chip rescanned within a few minutes. Nonetheless, we included the damaged chip image for analysis in this study. As a result, dataset A consisted of a total of 25 chips (8 biological samples by 3 technical replicates plus the damaged technical replicate for biological sample 4). Each chip contained 362,404 features on the array in the (x, y) coordinate system of size 602 × 602 features.

Affymetrix GeneChip arrays use 25-bp oligonucleotides to probe gene expression. Each gene or mRNA is represented by a probe set of 11–20 probe pairs of these oligonucleotides [6]. Each probe pair is composed of a PM probe and an MM probe created by changing the middle (13^th^) base of PM with the intent of measuring non-specific binding. The default for Affymetrix analysis software uses a measure of the difference in signal intensity between the PM and MM of probe pairs for each array. However, a few studies have suggested that the differences of PM-MM that intend to correct for non-specific binding are not always appropriate expression measurements [7-10]. Therefore, we demonstrated the performance of the proposed method for three types of measurements: intensity difference (PM-MM), PM-only intensity, and MM-only intensity. Each array used in this study contained 175,477 features for PM-only and MM-only measurements.

#### Experiment B

This experiment used a polycystic kidney disease knockout mouse model to compare gene expression in cystic kidneys from 8-week-old congenic *Pkd2*^-/-/pCAGGS-*PKD2 *^mice and control kidneys from *Pkd2*^+/+/pCAGGS-*PKD2 *^littermates. All mice used here were congenic (i.e., they were genetically identical except for different alleles at the *Pkd2 *locus) eliminating confounding effects of the genetic background. Kidneys from *Pkd2*^-/-/pCAGGS-*PKD2 *^mice and *Pkd2*^+/+/pCAGGS-*PKD2 *^controls were dissected 3–4 days postnatally. The tissues were homogenized in TRIzol reagent (Life Technologies) and RNA was extracted according to the manufacturer's protocol. The RNA was then purified using an RNeasy Mini kit (Qiagen Inc.) The total RNA samples from control and cystic kidneys were separately pooled and run on 3 Affymetrix GeneChip arrays per pooled RNA sample. After hybridization, images of the arrays were scanned by an Agilent GeneArray scanner (Agilent Technologies Co., Palo Alto, CA) and *.Cel files were generated by MAS 5.0 and used in this study. These Affymetrix arrays were run in the Affymetrix GeneChip Resource of the W.M. Keck Biotechnology Laboratory at Yale University. Dataset B contained a total of six chips for two pooled biological cases with three technical replicates. Each chip contained 409,600 features on the array in the (x, y) coordinate system of size 640 × 640 features.

#### Experiment C

This experiment used RNA samples collected from *Mus musculus *(C57Bl/6) mouse kidneys that had either undergone sham operation (control) or 25 min of ischemia (clamping of the renal artery) followed by 24 hours of reperfusion. The mice used in this experiment were anesthetized with 100 mg/kg ketamine and 10 mg/kg xylazine injected intraperitoneally and a midline incision was made for both sham and experimental animals. For bilateral ischemia/reperfusion (I/R), the left and right renal pedicles were clamped for 25 min using a vascular clamp (Fine Science Tools Inc., Foster City, CA). The sham-operated control mice were treated in the same way except that the renal pedicles were not clamped. The abdomen was covered with gauze moistened in phosphate buffered saline, and the mice were maintained at 37°C using a warming pad. At specified times the clamp was removed. At 12 hours post I/R, the mice were sacrificed and their kidneys were collected and homogenized in RNA-Stat (Tel-test Inc., Friendswood, TX). The total RNA samples from six control and ischemic kidneys were separately pooled and run on two different array platforms, a NIA15K cDNA spotted array and an Affymetrix oligonucleotide array. Images of the cDNA arrays were created on an Axon GenePix 4000A scanner and spot intensity data were obtained using GenePix Pro 3.0 software (Axon Instruments, Inc., Union City, CA); Affymetrix GeneChip images were generated and quantitated by MAS 5.0. These arrays were run in the Microarray Resource and the Affymetrix Resource of the W.M. Keck Biotechnology Laboratory at Yale University, respectively. Dataset C contained a total of six arrays with three technical replicates for each platform. Each Affymetrix chip contained 409,600 features on the array in the (x, y) coordinate system of size 640 × 640 features. Each cDNA spotted array was composed of 32 sectors on the slide, and each sector contained 552 features in the (x, y) coordinate system of size 23 × 24 features.

### Data preprocessing

Note that all datasets in these analyses were raw intensity measurements without treatment before analysis. Raw measurements were used because 1) data treatments, such as background subtraction or adjustment, would change the original behavior of measurements that interfered with the purpose of the proposed metric for assessing a chip quality and 2) the metric of untreated measurements was more valid when comparing reliability between arrays of the same type or cross platforms. Therefore, any treatment or adjustment of data was deemed inappropriate. Concerning measures of gene expression for Affymetrix chips, the literature has converged on using other measures, such as RMA [8] and dChip [9]. Those measures provide model-based summaries of gene expression for probe-level data rather than raw intensities, and future work will evaluate the performance of the presented method using model-based summary measurements of gene expression. However, estimation of spatial correlation at the raw-intensity level was our main goal and thus we considered the raw intensity value for each probe (i.e., feature) from a *.Cel file as a measurement unit and used it for analysis. Each *.Cel file provides a Cartesian coordinate for each feature. Similarly for spotted arrays we used raw fluorescent channel intensity for each spot without any manipulation of data.

### Statistical methods

Herein, we derive an approach that regresses intensity measurements on a third degree of polynomial response surface [11] of their microarray's Cartesian coordinates to calculate GEODEX as an indicator of the overall measurement reliability.

#### Methods for calculating a geography index

##### Primary model

For a given array, define *x *= X-axis microarray's Cartesian coordinate and *y *= Y-axis microarray's Cartesian coordinate. A third-degree polynomial response surface is defined by

*k *= *x *+ *y *+ *x*^2 ^+ *xy *+ *y*^2 ^+ *x*^3 ^+ *x*^2^*y *+ *xy*^2 ^+ *y*^3 ^+ *xy*^3 ^+ *x*^2^*y*^2 ^+ *x*^3^*y *+ *x*^2^*y*^3 ^+ *x*^3^*y*^2 ^+ *x*^3^*y*^3^,     (1)

where *k *is the raw intensity value of gene expression at (*x*, *y*). By raw intensity values we mean the intensity value for each probe (feature) from a *.Cel file output. Both PM and MM probes are considered for Affymetric GeneChip probe level data. Details of primary model selection, including goodness-of-fit of models and selection of influential factors on estimation, are given in subsequent sections.

We fit model (1) to all intensity measurements from an array to calculate the coefficient of determination, R^2^, ranged between 0 and 1, that represents the spatial variation in intensity measurements explained by the surface. If the array appears to have a random pattern, R^2 ^should be small (i.e., close to zero) because of the lack of fitness of the polynomial surface.

We define the value of 1-R^2 ^as the "geography index" that is a summary of array reliability based on spatial correlation and is denoted by GEODEX. We expect a higher value of 1-R^2 ^in agreement with the degree of geographic independence of intensity measurements based on their geographic locations on the surface of array. A higher value implies less spatial correlation between the intensity measurements and their physical locations on an array, meaning that the array is more reliable. R^2 ^was obtained by generalized regression models using the PROC GLM procedure implemented in SAS (SAS version 9.0; SAS, Cary, NC).

#### Goodness-of-fit of models

A polynomial surface is most suitable for assessing spatial variation of measurements in high dimensions [11]. An important issue in fitting polynomial models is the specification of the degree of polynomials. Obviously, the higher the polynomial degree, the better the fit of the model; however, models that include minimal parameters (i.e., coefficients of predictors in a model) are preferred. Herein, we consider only three representative polynomial models – linear, quadratic, and cubic curves, which include the products of variables and the powers of each variable. For instance, model (1) is a cubic polynomial response surface model with two coordinate indices, *x *and *y*. To exhibit the variant of goodness of fit by different models, we performed a step-forward variable selection procedure starting from a quadratic model using dataset A. All individual variables in the quadratic model were significant (i.e., p-values of F-test < 0.05). Hence, we extended our analysis to a cubic model. All variables in the cubic model were significant for most of the chips, and few variables were not significant for a few chips. Although the few variables were not individually significant, the full model itself was significant for all 25 arrays. For a cross comparison between chips, we needed a single fixed model that best fitted all 25 arrays to get relative GEODEX values across all chips, and thus we decided to retain all variables in the model. Furthermore, extended analyses with a quartic model did not result in substantial differences between the cubic model and the quartic model in terms of significant overlapping variables. Some of the parameter variables in the quartic model were insignificant. Using this forward-selection variable-searching procedure, we chose an intermediate (cubic) polynomial response model as our primary model (1) to calculate GEODEX. However, the power of variables needs not be restricted to three in a model to incorporate our proposed approach. We will elaborate in more detail regarding searching for optimal models [11] attempting to provide more prescription-type methods for non-statistical investigators.

#### Factors influencing the estimation of a geography index

Another concern in regression analysis would be finding key regressors for the measurements. Therefore, defining key factors that influence spatial variations is of great importance for estimating reliable GEODEX. We explored factors potentially responsible for spatial variations.

##### Block effects

Although global GEODEX estimated by model (1) assesses the physical reliability of each array, redundant variation by relatively small artifacts on an array may be neglected; however, this could be a vital factor that enhances overall spatial variation. Therefore, we introduced blocks (e.g., print-tip sectors) into model (1) as a potential regressor of block-dependent variability. This regressor restrains the contribution of within-blocks variation and exaggerates the contribution of between-blocks variation. To engage it in model (1), we first need to divide a whole microarray slide systematically into block units. For this study, each Affymetix™ chip was systematically divided into N_B _evenly spaced blocks that were sectored from left to right and top to bottom. These started from the top left corner when the manufacturing systems did not provide outputs of automatically assigned blocks or sectors. In two-color spotted microarray systems, multiple spots are simultaneously printed as a unit per print-tip on array glasses (i.e., hundred spots per print-tip). In this case there may be variation between print-tips. In fact, Yang et al. [12] noted that the standard deviation of intensity between print-tips could be large and increase the overall variation. In practice, print-tip units can be defined as blocks in analysis or users can define blocks based on their own judgment. In this study we used N_B _= 32 defined by print-tips. Analysis of variance can be used to determine whether there is evidence of significant block effects. To further elaborate this proposed approach, we hypothesize that there is significant evidence of block effects: blocks show different patterns associated with their geographic locations on an array and thus affect between-blocks variance. In particular, one would expect that inconsistency of blocks on the edges of arrays was substantial as a result of unstable hybridization around the edges of arrays. For these reasons, we declare blocks to be an inflational factor and include them in model (1) to reflect block-dependent variability. The block-dependent polynomial response surface is designed as follows:

*k *= *x *+ *y *+ *x*^2 ^+ *xy *+ *y*^2 ^+ *x*^3 ^+ *x*^2^*y *+ *xy*^2 ^+ *y*^3 ^+ *xy*^3 ^+ *x*^2^*y*^2 ^+ *x*^3^*y *+ *x*^2^*y*^3 ^+ *x*^3^*y*^2 ^+ *x*^3^*y*^3 ^+ *Block*_*k*_,     (2)

where *Block*_*k *_is a block indicator variable (i.e., *Block*_*k *_= 1, ..., N_B_, where N_B _= the number of defining blocks per chip) and is treated as a random variable. This model with an additional covariate performs robust local fits. In particular, it will be affected by small artifacts or a small geographic cluster of differentially expressed genes shown only in certain blocks, which are most likely outliers.

##### Blockwise geography index

*A *block-by-block analysis of individual chips can be used to classify sources of bias and to locate suspect regions of artifacts by detecting deviant blocks. To assess discrepancy among blocks, we computed a blockwise GEODEX per block. These local indices assist in the location of neighbors where small artifacts or unusual behaviors occur.

For a given *j*th block (print-tip or sector) *B*_*ij *_of array *i*, a local GEODEX can be calculated by regressing measurements within block *B*_*ij *_on the model

kBij
 MathType@MTEF@5@5@+=feaafiart1ev1aaatCvAUfKttLearuWrP9MDH5MBPbIqV92AaeXatLxBI9gBaebbnrfifHhDYfgasaacH8akY=wiFfYdH8Gipec8Eeeu0xXdbba9frFj0=OqFfea0dXdd9vqai=hGuQ8kuc9pgc9s8qqaq=dirpe0xb9q8qiLsFr0=vr0=vr0dc8meaabaqaciaacaGaaeqabaqabeGadaaakeaacqWGRbWAdaWgaaWcbaGaemOqai0aaSbaaWqaaiabdMgaPjabdQgaQbqabaaaleqaaaaa@3234@ = *x *+ *y *+ *x*^2 ^+ *xy *+ *y*^2 ^+ *x*^3 ^+ *x*^2^*y *+ *xy*^2 ^+ *y*^3 ^+ *xy*^3 ^+ *x*^2^*y*^2 ^+ *x*^3^*y *+ *x*^2^*y*^3 ^+ *x*^3^*y*^2 ^+ *x*^3^*y*^3^,     (3)

where kBij
 MathType@MTEF@5@5@+=feaafiart1ev1aaatCvAUfKttLearuWrP9MDH5MBPbIqV92AaeXatLxBI9gBaebbnrfifHhDYfgasaacH8akY=wiFfYdH8Gipec8Eeeu0xXdbba9frFj0=OqFfea0dXdd9vqai=hGuQ8kuc9pgc9s8qqaq=dirpe0xb9q8qiLsFr0=vr0=vr0dc8meaabaqaciaacaGaaeqabaqabeGadaaakeaacqWGRbWAdaWgaaWcbaGaemOqai0aaSbaaWqaaiabdMgaPjabdQgaQbqabaaaleqaaaaa@3234@ is the intensity level of gene expression at (*x*, *y*) within block *B*_*ij*_. This blockwise index, GEODEX_*Bij*_, represents the degree of block reliability of block *B*_*ij *_of chip *i*.

#### Assessing the validity of geography index as a predictor of reproducibility of arrays

Assuming that GEODEX values are correlated with inter-chip discrepancy, we use GEODEX as a measure of the lack of concordance for a pair of arrays. To assess the validation of this hypothesis, we calculate two references: one that provides the degree of similarity between two technical replicates (inter-chip discrepancy metric) and another that provides a summary of spatial correlation of two technical replicates (pairwise GEODEX). We suggest a convenient way of quantifying them for two technical replicates being compared. Briefly, consider two replicates, chip *i *and chip *j*, from the same biological specimen. A metric of inter-chip discrepancy between these two chips is the average relative deviation between two measurement replicates and explains the magnitude of discrepancy between these two chips. This metric can be calculated by

dij=1m∑l=1m|kil−kjl|(kil+kjl2),     (4)
 MathType@MTEF@5@5@+=feaafiart1ev1aaatCvAUfKttLearuWrP9MDH5MBPbIqV92AaeXatLxBI9gBaebbnrfifHhDYfgasaacH8akY=wiFfYdH8Gipec8Eeeu0xXdbba9frFj0=OqFfea0dXdd9vqai=hGuQ8kuc9pgc9s8qqaq=dirpe0xb9q8qiLsFr0=vr0=vr0dc8meaabaqaciaacaGaaeqabaqabeGadaaakeaacqWGKbazdaWgaaWcbaGaemyAaKMaemOAaOgabeaakiabg2da9maalaaabaGaeGymaedabaGaemyBa0gaamaaqahabaWaaSaaaeaadaabdaqaaiabdUgaRnaaBaaaleaacqWGPbqAcqWGSbaBaeqaaOGaeyOeI0Iaem4AaS2aaSbaaSqaaiabdQgaQjabdYgaSbqabaaakiaawEa7caGLiWoaaeaadaqadaqaamaalaaabaGaem4AaS2aaSbaaSqaaiabdMgaPjabdYgaSbqabaGccqGHRaWkcqWGRbWAdaWgaaWcbaGaemOAaOMaemiBaWgabeaaaOqaaiabikdaYaaaaiaawIcacaGLPaaaaaaaleaacqWGSbaBcqGH9aqpcqaIXaqmaeaacqWGTbqBa0GaeyyeIuoakiabcYcaSiaaxMaacaWLjaWaaeWaaeaacqaI0aanaiaawIcacaGLPaaaaaa@58D1@

where *K*_*il *_denotes the intensity value for the *l *th gene of chip *i *and *m *is the total number of features (spots) on chip *i*. Similarly, an average pairwise geography index can be given by

Gij=12(GEODEXi+GEODEXj),     (5)
 MathType@MTEF@5@5@+=feaafiart1ev1aaatCvAUfKttLearuWrP9MDH5MBPbIqV92AaeXatLxBI9gBaebbnrfifHhDYfgasaacH8akY=wiFfYdH8Gipec8Eeeu0xXdbba9frFj0=OqFfea0dXdd9vqai=hGuQ8kuc9pgc9s8qqaq=dirpe0xb9q8qiLsFr0=vr0=vr0dc8meaabaqaciaacaGaaeqabaqabeGadaaakeaacqWGhbWrdaWgaaWcbaGaemyAaKMaemOAaOgabeaakiabg2da9maalaaabaGaeGymaedabaGaeGOmaidaaiabcIcaOiabdEeahjabdweafjabd+eapjabdseaejabdweafjabdIfaynaaBaaaleaacqWGPbqAaeqaaOGaey4kaSIaem4raCKaemyrauKaem4ta8KaemiraqKaemyrauKaemiwaG1aaSbaaSqaaiabdQgaQbqabaGccqGGPaqkcqGGSaalcaWLjaGaaCzcamaabmaabaGaeGynaudacaGLOaGaayzkaaaaaa@4B62@

where *GEODEX*_*i *_is the global GEODEX of chip *i*. Note that *GEODEX*_*i *_in equation (5) can be altered by functional statistics of blockwise GEODEX (*GEODEX*_*Bi*_) on chip *i*. For example, maximum/minimum blockwise GEODEX (*max*(*GEODEX*_*Bi*_)/*min*(*GEODEX*_*Bi*_)) can replace *GEODEX*_*i*_. The *q*th percentile of blockwise GEODEX values (*Q*_*q*_(*GEODEX*_*Bi*_)) can also replace *GEODEX*_*i*_. Then we can draw a scatter plot with *G*_*ij *_on the X-axis and *d*_*ij *_on Y-axis, showing the relationship between the concordance of measurement replicates and the average spatial correlation. Presumably, lower inter-chip discrepancy implies high reproducibility of technical replicates and high pairwise GEODEX implies high reliability of technical replicates on average; thus, pairwise GEODEX is negatively associated with inter-chip discrepancy. On the basis of this inter-relationship, pairwise GEODEX can be used as a predictor of the reproducibility of arrays in microarray experiments.

## Authors' contributions

KK developed the method and performed analyses. GPP, TMB, and SB contributed to discussion. KES performed microarray experiment A in the manuscript. DBA conceived and mentored the project. All authors contributed to the writing of the manuscript. The final manuscript has been reviewed and approved by all authors.

## Figures and Tables

**Table 1:** Descriptive statistics of measurements of individual chips in experiment A and summaries of resulting GEODEX. Five chips (9, 12, 14, 15, and 23) in boldface below consistently have the lowest GEODEX irrespective of the number of blocks.

^1^Chip ID	^2^Mean	^3^SD	^4^CV	^5^GEODEX^[1]^	^6^GEODEX^[2]^		
					
					N_B _= 9	N_B _= 36	N_B _= 144
1	298.24	562.15	188.48	0.9948	0.9932	0.9901	0.9863
2	306.96	588.32	191.65	0.9962	0.9950	0.9927	0.9896
3	387.64	799.63	206.27	0.9934	0.9920	0.9890	0.9853
4	309.45	581.16	187.79	0.9951	0.9935	0.9906	0.9868
5	398.48	847.48	212.67	0.9943	0.9929	0.9901	0.9867
6	364.01	822.76	226.02	0.9943	0.9925	0.9892	0.9853
7	403.05	866.67	215.02	0.9925	0.9907	0.9873	0.9831
8	302.11	854.81	282.94	0.9965	0.9957	0.9944	0.9922
9	372.76	818.37	219.54	**0.9907**	**0.9886**	**0.9847**	**0.9797**
10	482.55	961.08	199.16	0.9932	0.9916	0.9885	0.9847
11	290.04	563.84	194.39	0.9948	0.9934	0.9905	0.9869
12	288.50	687.20	238.19	**0.9685**	**0.9668**	**0.9624**	**0.9570**
13	343.23	663.09	193.18	0.9941	0.9929	0.9902	0.9868
14	290.12	610.07	210.27	**0.9903**	**0.9887**	**0.9856**	**0.9814**
15	236.84	482.26	203.61	**0.9892**	**0.9876**	**0.9843**	**0.9799**
16	261.13	833.62	319.22	0.9976	0.9969	0.9958	0.9940
17	501.95	1014.49	202.10	0.9909	0.9893	0.9863	0.9824
18	309.42	624.82	201.93	0.9938	0.9923	0.9893	0.9856
19	261.58	473.18	180.88	0.9938	0.9926	0.9898	0.9861
20	328.96	668.14	203.10	0.9962	0.9946	0.9917	0.9879
21	281.70	543.55	192.95	0.9952	0.9939	0.9914	0.9881
22	356.03	692.10	194.39	0.9953	0.9939	0.9912	0.9878
23	367.10	840.50	228.95	**0.9901**	**0.9883**	**0.9849**	**0.9804**
24	395.99	776.34	196.05	0.9959	0.9948	0.9922	0.9891
25	293.60	617.81	210.42	0.9943	0.9930	0.9903	0.9867

1. Chip ID is the identification number of chip.

2. MEAN is the average of intensity measurements of gene expression for each chip.

3. SD is the standard deviation of intensity measurements of gene expression for each chip.

4. CV is the coefficient of variation of intensity measurements of gene expression for each chip.

5. GEODEX^[1] ^is a geography index calculated by model (1).

6. GEODEX^[2] ^is a geography index calculated by model (2) (N_B _= the number of defining blocks per chip).

**Table 2:** Descriptive statistics and resulting GEODEX of a simulation study.

^1^Chip ID	^2^MEAN	^3^SD	^4^CV	^5^GEODEX^[1]^	^6^GEODEX^[2]^
Sample	298.2445	562.1518	1.88	0.99493965	0.99015619
Scenario 1	298.245	562.1518	1.88	0.9949455	0.99015956
Scenario 2	295.4852	562.2858	1.90	0.99422959	0.98896773
Scenario 3	305.9073	574.8667	1.87	0.99588081	0.9852998
Scenario 4	367.2101	934.5144	2.54	0.97747889	0.8120757
Scenario 5	1056.8657	7701.3605	7.28	0.95547486	0.67372248
Scenario 6	4609.2052	43392.8549	9.41	0.95409713	0.66822548
Scenario 7	8927.8288	86801.9967	9.72	0.95396446	0.66776258
Scenario 8	13246.4524	130212.341	9.82	0.95392095	0.66761398

1. Chip ID is the identification number of simulated chip.

2. MEAN is the average of intensity measurements of gene expression for each chip.

3. SD is the standard deviation of intensity measurement of gene expression for each chip.

4. CV is the coefficient of variation of intensity measurements of gene expression for each chip.

5. GEODEX^[1] ^is a geography index calculated by model (1).

6. GEODEX^[2] ^is a geography index calculated by model (2) (N_B _= 36).
